# Early Pulmonary Fibrosis-like Changes in the Setting of Heat Exposure: DNA Damage and Cell Senescence

**DOI:** 10.3390/ijms25052992

**Published:** 2024-03-05

**Authors:** Tong Hou, Jiyang Zhang, Yindan Wang, Guoqing Zhang, Sanduo Li, Wenjun Fan, Ran Li, Qinghua Sun, Cuiqing Liu

**Affiliations:** 1 School of Public Health, Zhejiang Chinese Medical University, Hangzhou 310053, China; houtong1207@163.com (T.H.); zjy@zcmu.edu.cn (J.Z.); 15715732579@163.com (Y.W.); gqzhang2015@126.com (G.Z.); sanduoli0@163.com (S.L.); fanwenjun0110@163.com (W.F.); 20171007@zcmu.edu.cn (R.L.); qhsun@zcmu.edu.cn (Q.S.); 2 Zhejiang International Science and Technology Cooperation Base of Air Pollution and Health, Hangzhou 310053, China

**Keywords:** heat exposure, lung fibrosis, DNA damage, senescence, cGAS–STING pathway

## Abstract

It is well known that extreme heat events happen frequently due to climate change. However, studies examining the direct health impacts of increased temperature and heat waves are lacking. Previous reports revealed that heatstroke induced acute lung injury and pulmonary dysfunction. This study aimed to investigate whether heat exposure induced lung fibrosis and to explore the underlying mechanisms. Male C57BL/6 mice were exposed to an ambient temperature of 39.5 ± 0.5 °C until their core temperature reached the maximum or heat exhaustion state. Lung fibrosis was observed in the lungs of heat-exposed mice, with extensive collagen deposition and the elevated expression of fibrosis molecules, including transforming growth factor-β1 (TGF-β1) and Fibronectin (Fn1) (*p* < 0.05). Moreover, epithelial–mesenchymal transition (EMT) occurred in response to heat exposure, evidenced by E-cadherin, an epithelial marker, which was downregulated, whereas markers of EMT, such as connective tissue growth factor (CTGF) and the zinc finger transcriptional repressor protein Slug, were upregulated in the heat-exposed lung tissues of mice (*p* < 0.05). Subsequently, cell senescence examination revealed that the levels of both senescence-associated β-galactosidase (SA-β-gal) staining and the cell cycle protein kinase inhibitor p21 were significantly elevated (*p* < 0.05). Mechanistically, the cGAS–STING signaling pathway evoked by DNA damage was activated in response to heat exposure (*p* < 0.05). In summary, we reported a new finding that heat exposure contributed to the development of early pulmonary fibrosis-like changes through the DNA damage-activated cGAS–STING pathway followed by cellular senescence.

## 1. Introduction

Recently, the elevation of global temperatures due to climate change has resulted in an increase in the frequency and intensity of heat waves, contributing to the spread of health hazards [1]. According to the Intergovernmental Panel on Climate Change (IPCC), the current global temperature is about 1.1 °C higher than pre-industrial temperatures and will continue to increase by 1.5 °C until 2052 [2,3]. The abnormal rise in body temperature during heat exposure could damage multiple organs, and could cause heat stroke, or even death [4]. The mortality rate for classic heatstroke in intensive care in hospitals is as high as 63.2% [5]. Considering climate change and the large population of outdoor workers or firefighters, it is urgent to explore the adverse effects of high temperatures on health.

A retrospective cohort study in Shandong, China, confirmed that lifetime summer heat exposure is significantly associated with a reduction in lung function in young adults [6]. Pulmonary fibrosis and chronic obstructive pulmonary disease (COPD) are both chronic lung diseases that are characterized by a progressive decline in lung function [7]. Studies have found that older people or people with COPD are at a higher risk of adverse health effects after heat exposure. For example, a study based on 12 cities in the United States estimated that high temperatures increase deaths from COPD by as much as 25% [8]. In addition, an observational study also showed that each 10 °F (5.6 °C) increase in daily temperature was associated with a 4.3% increase in same-day emergency hospitalizations for respiratory diseases [9]. Lung fibrosis is a progressive, chronic lung disease characterized by interstitial inflammation, disrupted alveolar structure, fibroblast proliferation and the excessive accumulation of extracellular matrix (ECM) [10]. Pulmonary interstitial inflammation is usually an early event in the development of lung fibrosis [11]. A new study suggested that heat exposure induced lung inflammation in mice [12]. However, whether acute heat exposure could lead to lung fibrosis and the relevant mechanisms remain unknown. 

DNA damage increases significantly after acute heat stress exposure, especially in hot humid and hot dry climates [13]. As recognized, the integrity of DNA, as an essential carrier of genetic information, is critical for normal cellular function and organismal development [14]. However, DNA at high temperatures faces disrupted structure and function, leading to DNA damage, including DNA double-strand breaks and base damage [15]. The aberrant presence of double-stranded DNA in the cytoplasm could be detected by cyclic GMP-AMP (cGAMP) synthase (cGAS), which catalyzes the release of an intrinsic immune response via the binding of cGAMP to a stimulator of interferon gene (STING) dimers on the endoplasmic reticulum [16,17,18]. These responses help to maintain intracellular homeostasis and promote DNA repair, senescence, and the clearance of damaged cells [19]. Conversely, unbalanced cGAS–STING signaling has considerable relevance in fibrotic diseases, pulmonary hemorrhage, and lung dysfunction [20]. Similarly, prolonged overactivation may lead to the development of a chronic inflammatory state and accelerate the aging process.

DNA damage is a well-recognized feature of genomic instability and is a hallmark of ageing [21]. Cellular senescence is a stress-induced irreversible cell cycle arrest characterized by the increased expression of senescence-associated β-galactosidase (SA-β-gal) and p21^CIP1^ activation [22]. It has been demonstrated that the accumulation of senescent cells contributes to age-related diseases, including fibrosis [23]. As cells enter the senescence state, they may exhibit a number of phenotypic features associated with EMT, such as altered morphology, a decreased expression of epithelial markers and an increased expression of mesenchymal markers, which are critical molecules required in the fibrosis process [24]. In addition, the expression of intracellular cell adhesion molecules (such as E-cadherin) is altered, leading to a decrease in intercellular adhesion, which allows the epithelial cells to dissociate and gradually migrate into the mesenchymal regions. During this process, cells may also release and rearrange extracellular matrix components to provide support for cell migration and fibrosis [25]. Age-related findings have now been reported in idiopathic pulmonary fibrosis (IPF), and 25% of familial IPF cases are associated with mutations affecting telomere maintenance [26,27]. Fibrotic remodeling during cellular senescence not only impairs regenerative function but also affects the surfactant function of ATII cells. Dysfunctional alveolar type II epithelial cells (ATII) not only fail to maintain physiological lung regeneration, but also promote abnormal epithelial–mesenchymal crosstalk [28]. Systemic sclerosis-associated pulmonary fibrosis (SSc-PF) is the leading cause of death in systemic sclerosis, accounting for approximately 55% of deaths. This study found that TGFβ3 is a down-regulated gene in SSc-transplanted lungs, which also contradicts previous findings. However, it has also been suggested that TGFβ3 negatively regulates the fibrotic response and accelerates wound healing without scarring [29]. Current therapeutic interventions designed to target the inhibition of the transforming growth factor-β (TGF-β) signaling pathway have shown promising efficacy in ameliorating pulmonary fibrosis [30]. DNA damage and cellular stress trigger the activation and migration of inflammatory and fibroblast cells, promoting the fibrotic process. In addition, DNA damage may cause cells to enter a state of cell cycle arrest, limiting their ability to proliferate and repair. Thus, we hypothesize that DNA damage-mediated cellular senescence may contribute to heat exposure-induced fibrosis in the lung.

Therefore, in this study, we investigated the pathological process of pulmonary fibrosis in heat-exposed mice and tried to explain the mechanism from the perspective of DNA damage/the cGAS–STING signaling pathway/the cellular senescence axis. This study draws attention to the harmful effects of heat exposure on the lungs and contributes to an in-depth understanding of the mechanisms by which hyperthermia affects human health, and is expected to provide some theoretical basis for clinical treatment. 

## 2. Results

### 2.1. Heat Exposure-Induced Early Pulmonary Fibrosis-like Changes in Mice

**Figure 1:**
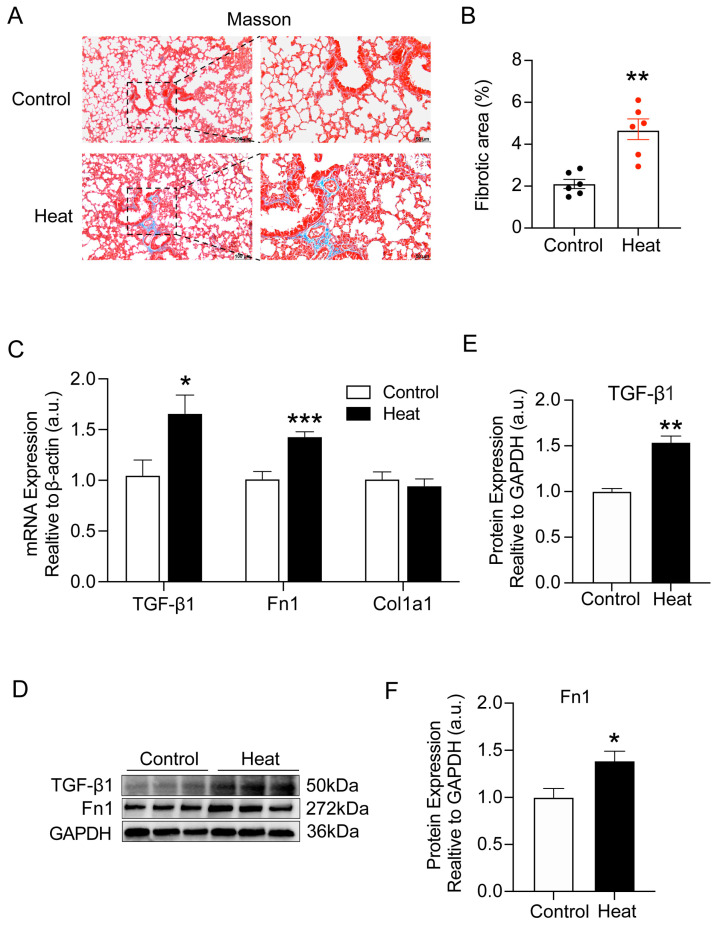
Heat exposure-induced early pulmonary fibrosis-like changes in mice. (**A**,**B**) Representative images and quantitative analysis of Masson’s staining of the lung tissue. Scale bar = 100 μm. Locally magnified images of lung tissue sections (scale bar = 50 µm). (**C**) Gene expression of TGF-β1, Fn1, and Col1a1 in lung tissues was quantitated using qRT-PCR. (**D**–**F**) Western blot for lung TGF-β1 and Fn1 protein and quantification analysis. (*n* = 7) (* *p* < 0.05, ** *p* < 0.01 and *** *p* < 0.001 vs. Control).

Firstly, we found that there was no significant difference in the lung weight (*p* = 0.195) and lung coefficient (*p* = 0.096) between the control and heat groups in mice. To further assess whether heat exposure caused pulmonary collagen deposition in mice, we performed Masson’s trichrome staining of lung tissues. As shown in Figure 1A,B, significant collagen deposition was observed in the lung tissues of mice after heat exposure (*p* < 0.01). Meanwhile, we also examined the gene and protein expression of lung fibrosis-related molecules (TGF-β1, Fn1, and Col1a1). Undoubtedly, both gene and protein levels of TGF-β1 and Fn1 appeared significantly elevated (*p* < 0.05, Figure 1C–F), further confirming the occurrence of early pulmonary fibrosis-like changes in mice after heat exposure.

### 2.2. Heat Exposure-Induced EMT in Lung Tissue of Mice

**Figure 2:**
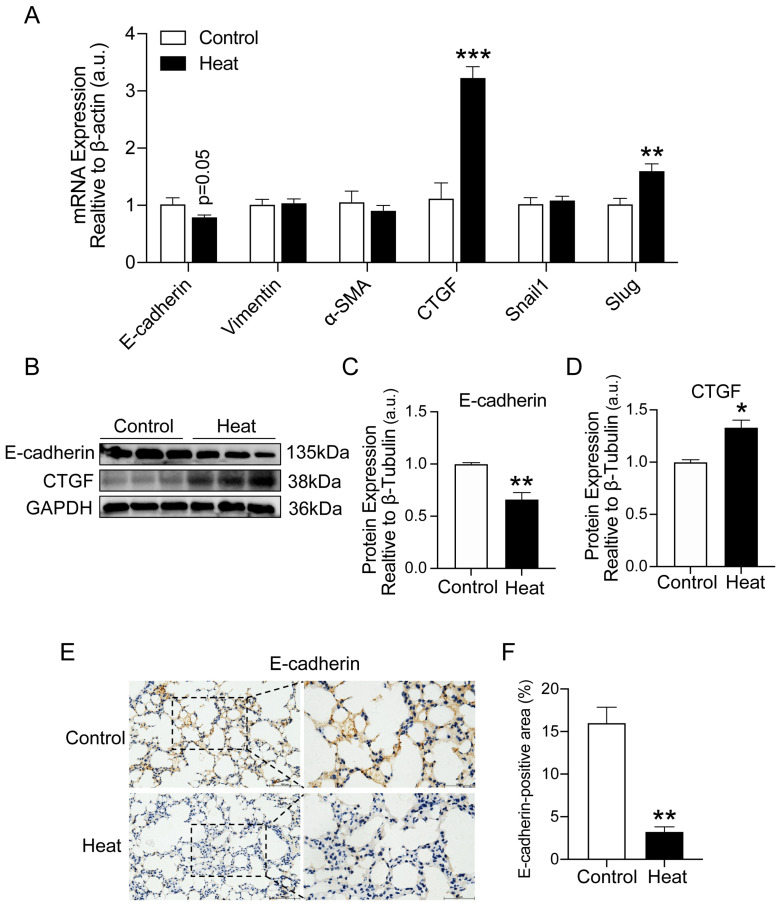
Heat exposure-induced EMT in the lung tissue of mice. (**A**) Gene expressions of E-cadherin, Vimentin, α-SMA, CTGF, Snail1 and Slug in the lung tissues were quantitated using qRT-PCR. (**B**–**D**) Western blot for lung E-cadherin and CTGF protein and quantification analysis. (**E**,**F**) Lung E-cadherin was measured using IHC and quantification analysis. Scale bar = 100 μm. Locally enlarged images of lung tissue sections (scale bar = 50 µm). (*n* = 7) (* *p* < 0.05, ** *p* < 0.01 and *** *p* < 0.001 vs. Control).

Epithelial–mesenchymal transition (EMT) is involved in fibrotic development. To further assess the effect of heat exposure on lung fibrosis, we examined the expression of EMT-related genes (E-cadherin, Vimentin, α-SMA, CTGF, Snail1, Slug). As shown in Figure 2A, E-cadherin, one of the epithelial markers, was downregulated in the lungs of heat-exposed mice (*p* = 0.05). By contrast, the expressions of connective tissue growth factor CTGF (*p* < 0.001) and the transcription factor Slug (*p* < 0.01) were elevated after heat exposure. Similarly, the protein expression of E-cadherin (*p* < 0.01) and CTGF (*p* < 0.05) showed consistent changes (Figure 2B–D). Furthermore, we also examined the protein expression of E-cadherin (*p* < 0.01) by immunohistochemical experiments and found that the expression was significantly reduced in the lung tissues of mice after heat exposure compared with the control group (Figure 2E,F), verifying the occurrence of EMT in the lung tissues of mice.

### 2.3. Heat Exposure-Induced Lung Cellular Senescence in Mice

**Figure 3:**
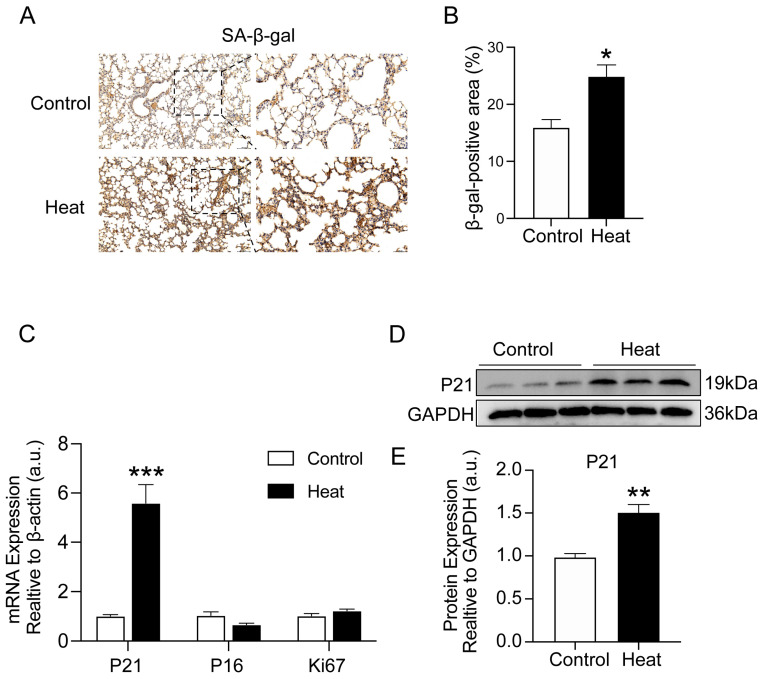
Heat exposure-induced lung cellular senescence in mice. (**A**,**B**) Lung β-gal was measured using IHC and quantification analysis. Scale bar = 100 μm. Locally enlarged images of lung tissue sections (scale bar =50 µm). (**C**) Gene expressions of P21, P16, and Ki67 in the lung tissues were quantitated using qRT-PCR. (**D**,**E**) Western blot for the lung P21 protein and quantification analysis. (*n* = 7) (* *p* < 0.05, ** *p* < 0.01 and *** *p* < 0.001 vs. Control).

Cellular senescence is now considered an important driving mechanism for chronic lung diseases, particularly pulmonary fibrosis [23]. To further explore whether heat exposure induced cellular senescence in the lung tissues of mice, we examined the protein expression of SA-β-gal. As shown in Figure 3A,B, the staining area in the lung tissues was significantly increased after heat exposure compared with the control group (*p* < 0.05). Similarly, both the gene (*p* < 0.001) and protein (*p* < 0.01) expression of P21, the marker of senescence, appeared significantly upregulated (Figure 3C–E). These results further identified that heat exposure induced lung cellular senescence in mice.

### 2.4. Heat Exposure-Induced DNA Damage and cGAS–STING Pathway Activation

**Figure 4:**
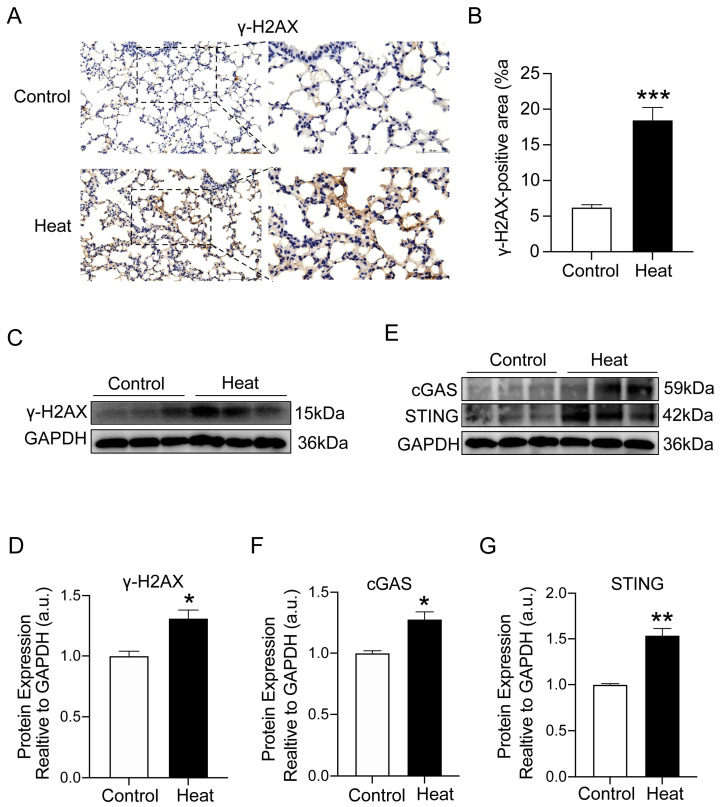
Heat exposure-induced DNA damage and cGAS–STING pathway activation. (**A**,**B**) Lung γ-H2AX was measured using IHC and quantification analysis. Scale bar = 100 μm. Locally enlarged images of lung tissue sections (scale bar = 50 µm). (**C**,**D**) Western blot for lung γ-H2AX protein and quantification analysis. (**E**–**G**) Western blot for lung cGAS and STING protein and quantification analysis. (*n* = 7) (* *p* < 0.05, ** *p* < 0.01 and *** *p* < 0.001 vs. Control).

DNA damage has been shown to be an essential cause of cellular senescence. To assess DNA damage induced by heat exposure, we examined the protein expression of γ-H2AX, a marker of DNA damage. Clearly, IHC staining (*p* < 0.001) and protein (*p* < 0.05) examination demonstrated that the expression of γ-H2AX was significantly elevated in the lung tissues of mice after heat exposure compared to the control group (Figure 4A–D). Accumulated DNA damage usually triggers the activation of the cGAS–STING pathway, which further regulates the onset of cellular senescence. To verify whether cellular senescence induced by heat exposure is related to the cGAS–STING pathway, we examined the protein levels of cGAS (*p* < 0.05) and STING (*p* < 0.01) in mouse lung tissues and observed a significant elevation compared with the control group (Figure 4E–G). These results indicated that heat exposure-induced DNA damage in lung tissue may be induced by activating the cGAS–STING signaling pathway.

## 3. Discussion

As an extension of the investigation into extreme heat-induced lung injury (unpublished), this present study further examined the adverse effects in the lungs of heat-exposed mice using a high-temperature exposure system. To our knowledge, it is the first study to demonstrate that heat exposure-induced early pulmonary fibrosis-like changes and DNA damage-evoked senescence via cGAS–STING pathway activation may be the underlying mechanism.

Heat exposure has seriously affected the quality of human life, posing a major threat to daily mortality rates and inducing a considerable healthcare burden [31]. Across all study countries (including 732 sites in 43 countries), 37.0% (range 20.5–76.3%) of warm-season heat-related deaths during 1991–2018 were attributable to increased heat exposure due to warming [32]. Studies in different populations have confirmed that heat exposure is associated with significant lung damage, such as asthma and chronic obstructive pulmonary disease (COPD) [33,34,35,36]. At ambient temperatures of 39.5 ± 0.2 °C, mice can reach a core temperature of 43 °C, with impaired respiratory function. Meanwhile, lung tissue is severely damaged, including alveolar collapse, vascular congestion, alveolar hemorrhage, interstitial thickening and neutrophil infiltration [12]. Pulmonary fibrosis is characterized by the abnormal proliferation and deposition of collagen fibers in the lung tissue, leading to impaired lung function. It has now been found that environmental factors may be involved [37]. We first found that the mice showed lung damage, with not only severe damage to the alveolar structure under heat exposure conditions, but also a large amount of collagen deposition in the lung tissue, as confirmed using Masson’s staining. Therefore, we considered the possibility of fibrosis of the lung tissue. Recognized fibrosis-related genes, such as TGF-β1, Fn1, and Col1a1, are closely associated with fibrotic cell dedifferentiation and collagen accumulation [38,39]. In the present study, we found no significant expression differences in Col1a1 expression, but the expressions of TGF-β1 and Fn1 were dramatically elevated in the lung tissues of heat-exposed mice. In addition, EMT is a biological process by which specific procedures transform epithelial cells into cells with a mesenchymal phenotype. This plays a vital role in a variety of fibrotic diseases [40]. High expression of Slug leads to the attenuation of intercellular adhesion and promotes cell separation and motility. CTGF is an essential extracellular matrix protein with functions such as the regulation of cell adhesion, cell migration and collagen synthesis [41]. Our results revealed that inducible molecules such as Slug and CTGF were obviously elevated in the mice’s lungs after heat exposure. By contrast, E-cadherin, an epithelial marker, was downregulated. These observations suggest that EMT is involved in the process of heat exposure-induced early pulmonary fibrosis-like changes. Notably, the above evidence supports the possibility that Slug and CTGF may be regulated by TGF-β, leading to the upregulation of expression, where Slug inhibits the expression of epithelial signature genes such as E-calmodulin and promotes the expression of mesenchymal signature genes such as Fn1. This leads to alterations in the morphology and function of the cells, which progressively lose epithelial features and acquire mesenchymal features. CTGF, on the other hand, complexly promotes cellular migration and invasion, and is involved in the alteration of extracellular matrix components. It alters the cellular environment by inducing the secretion and rearrangement of molecules such as Fn1, which supports cell migration and fibrosis. Thus, this evidence demonstrates that with the induction of EMT, early fibrosis-like changes occur in mouse lungs during heat exposure.

A recent study has found that heat stimulation at 40 °C for 72 h disrupts cellular system homeostasis, which in turn accelerates cellular ageing [42]. It has been found that heat shock could significantly reduce the proliferation and differentiation capacity of bone marrow mesenchymal stem cells and induce premature senescence [43,44]. In this study, the increased histochemical staining of SA-β-gal, a marker of cellular senescence, revealed pronounced senescence in the heat-exposed mouse lung tissue. Senescence is a complex, multifaceted process often accompanied by an inability to re-enter the cell cycle in response to mitotic stimuli [45]. The primary function of P21 is to inhibit cell cycle progression. It binds to and inhibits the activity of cell cycle-dependent kinases (e.g., CDK2, CDK4, and CDK6, etc.), thereby preventing the cell from entering the S-phase and the G2/M-phase and causing the cell cycle to be blocked in the G1-phase. P21, a cell cycle-related factor, is activated earlier upon engaging in senescence [46,47]. Recent reports have revealed that P21 expression is upregulated in the lungs of IPF patients compared to donor lungs [48,49]. Correspondingly, P21 was significantly overexpressed in response to heat exposure, further indicating that heat exposure aggravated cellular senescence in the mouse lung tissue. 

DNA damage is a condition that results in impaired cellular function, such as DNA double-strand breaks (DSBs) and DNA base damage. When a cell experiences severe DNA damage, it often experiences cell cycle block and the activation of DNA repair mechanisms to ensure the proper repair of the DNA damage. However, when DNA damage is excessive or cannot be repaired entirely, cells can enter a state of permanent cell cycle block, known as senescence [21]. There is accumulating evidence that DNA damage affects the majority of aging phenotypes, making it an important pathogenetic factor in senescence [50]. It has been found that excessive oxidative stress and DNA damage in the respiratory tract may contribute to the process of EMT in primary human small airway epithelial cells, and can lead to cellular changes associated with decreased respiratory function [51]. DNA damage activates the DNA damage response (DDR) pathway mainly in the form of DSBs, for which phosphorylated H2AX (γ-H2AX) is a sensitive marker. During senescence, the cell’s DNA repair capacity gradually decreases. This means that the accumulation of DNA damage increases, leading to the increased formation of γ-H2AX [52]. Our observations of elevated γ-H2AX expression in lung tissues from heat-exposed mice provided further evidence that cellular senescence may be involved in DNA damage. cGAS is a DNA sensor that recognizes intracellular DNA damage and subsequently activates STING to exert its immune function. Studies have shown that the inhibition of STING expression suppresses TGF-β1-induced fibroblast proliferation, differentiation and migration, as well as collagen production, suggesting that cGAS–STING signaling is involved in fibroblast activation [53]. This is consistent with the results of the present study. In view of the fact that cellular senescence is a key pathological event in the fibrotic process, the present study draws the preliminary conclusion that the activation of the cGAS–STING signaling pathway by DNA damage may be the mechanism involved in heat exposure-induced pulmonary fibrosis in mice. Thus, it is apparent that our findings express the roles and relationships of DNA damage, senescence and EMT in heat exposure-induced early pulmonary fibrosis-like changes in mice. This elucidates firstly that heat exposure induces the onset of DNA damage in mouse lungs and secondly that the sustained DNA damage will eventually further contribute to the process of lung cell senescence to EMT, which ultimately causes early pulmonary fibrosis-like changes.

## 4. Materials and Methods

### 4.1. Animals

All 9-week-old C57BL/6N male mice in this experiment were obtained from Beijing Vital River Laboratory Animal Technology Co., Ltd. (Beijing, China). The mice were housed under a 12 h light/dark cycle in a temperature-controlled room (22 ± 2 °C) with unrestricted access to an abundant chow diet and water. The mice were acclimatized for one week before formal experiments. The animal protocols were fully approved by the Animal Experimentation Committee of Zhejiang Chinese Medical University.

### 4.2. Experimental Design

Twenty mice were randomly divided into two groups, including the control (Control, 22 ± 1 °C) and the heat-exposed groups (Heat, 39.5 ± 0.5 °C). The heat exposure lasted for ~2.5 h when the mice reached the maximum core body temperature or heat exhaustion state. The initial humidity of the heating chamber was approximately 50–60%, which decreased as the temperature in the chamber increased. All mice were restricted from drinking water during the exposure period. At the end of the heat exposure, the mice were transferred to room-temperature conditions for a 2 h recovery phase until their core temperature decreased to the minimum level at which the inflammatory response and damage to the body is more intense [54,55]. The experimental design followed the principle of randomized control, and the control mice were given the same treatment except that they were not exposed to heat. The mouse lung tissue was completely stripped out and transferred rapidly to liquid nitrogen or 4% paraformaldehyde for subsequent gene, protein, and staining analysis.

### 4.3. Masson’s Staining

Mouse lung tissues were washed three times with PBS after the mice were sacrificed and then immediately transferred to 4% formaldehyde for fixation for 24 h. The lung samples were dehydrated with graded concentrations of ethanol and xylene, and the tissues were embedded in paraffin wax after being trimmed to the appropriate size. Slices measuring 5 μm were collected for Masson’s staining, where blue represents collagen fibers, red represents muscle fibers and blue–brown represents nuclei. Images were evaluated for collagen deposition levels and lung fibrosis using Image J software version 1.53.

### 4.4. Immunohistochemistry (IHC)

The 5 μm slices were heat-repaired with sodium citrate buffer (pH = 6), followed by a 15 min immersion in 3% hydrogen peroxide without light. After a 60 min blocking period with 10% goat serum, E-cadherin and SA-β-gal (both at a 1:100 dilution ratio) were used for overnight incubation. The color was visualized with DAB (9018, ZSGB-Bio, Beijing, China) after incubation with a (1:100 dilution ratio) secondary antibody. Image capture was performed with a light microscope.

### 4.5. Quantitative Real-Time PCR

The fresh lung tissue was placed in a TRIZOL reagent kit (9108, TaKaRa, Tokyo, Japan) and RNA was isolated after the tissue was thoroughly homogenized according to the manufacturer’s instructions. A PrimeScript RT Master Mix kit (6210, TaKaRa, Tokyo, Japan) was used to ensure that the RNA was reverse-transcribed into cDNA. The mRNA levels were detected using a SYBR Green Mix (A25742, Thermo, Waltham, MA, USA), all data were normalized by β-actin mRNA, and 2^−ΔΔCT^ methods were used to calculate the relative expression levels. The primer sequences are provided in Table 1.

### 4.6. Western Blot

Lung tissue was added to an RIPA lysate (AR0102, Boster, Wuhan, China) premixed with PMSF and a phosphatase inhibitor (ST506, Beyotime, Shanghai, China) and homogenized thoroughly. It was then placed on ice for 30 min and centrifuged at 4 °C and 12,000 rpm for 10 min to obtain the proteins. These proteins were assayed for concentration using a BCA kit (P0010S, Beyotime, Shanghai, China) and mixed with a loading buffer (WB2001, NCM Biotech, Suzhou, China) to make protein samples. After SDS-PAGE gel electrophoresis, the proteins were separated according to their molecular weight and size. After the membrane transfer operation, the protein samples were enriched on the PVDF membrane. Subsequently, the PVDF membrane containing the target proteins was blocked with a 5% BSA solution and sequentially incubated with primary and secondary antibodies. Finally, images were captured on a gel imaging system in conjunction with ECL (P10060, NCM Biotech, Suzhou, China) reagents. The antibodies are provided in Table 2.

### 4.7. Statistical Analysis

All data were presented as the mean ± standard error of the mean (SEM) and compared using the Student’s *t*-test. All data were analyzed at least three times. GraphPad Prism software version 9.3 was used for statistical analyses, and *p* < 0.05 was regarded as statistically significant.

## 5. Conclusions

**Figure 5:**
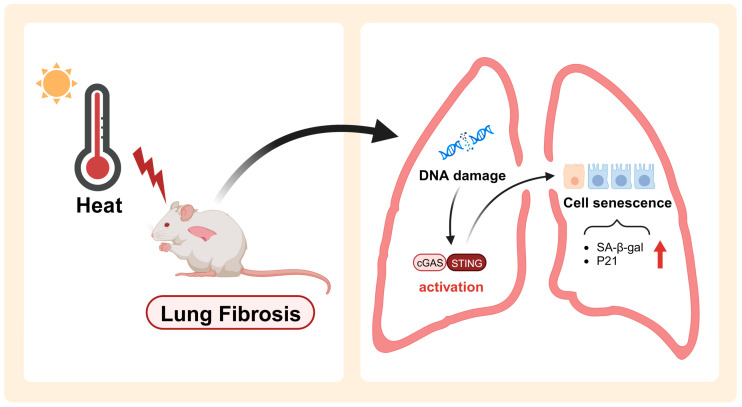
Schematic diagram of the mechanisms underlying heat exposure-induced early pulmonary fibrosis-like changes. Heat exposure induced DNA damage in mice lung tissues, which subsequently led to the activation of the cGAS–STING signaling pathway, further inducing lung cell senescence and ultimately leading to the development of early pulmonary fibrosis-like changes.

In summary, we first identified that DNA damage occurred in the lungs of mice under heat exposure, which may subsequently lead to the activation of the cGAS–STING signaling pathway, contributing to lung tissue senescence and, ultimately, early pulmonary fibrosis-like changes (Figure 5). Our findings may shed light on the health risks posed to workers in heat settings, offer guidelines for policy making, and provide clues for the therapeutic targets for intervention in heat-induced health hazards.

## Figures and Tables

**Table 1:** Sequences of qPCR primers.

Gene	Forward Primer	Reverse Primer
P16	CGCAGGTTCTTGGTCACTGT	TGTTCACGAAAGCCAGAGCG
Ki67	ATCATTGACCGCTCCTTTAGGT	GCTCGCCTTGATGGTTCCT
Snail1	CACACGCTGCCTTGTGTCT	GGTCAGCAAAAGCACGGTT
Slug	TGGTCAAGAAACATTTCAACGCC	GGTGAGGATCTCTGGTTTGGTA
CTGF	GGGCCTCTTCTGCGATTTC	ATCCAGGCAAGTGCATTGTA
E-cadherin	CAGGTCTCCTCATGGCTTTGC	CTTCCGAAAAGAAGGCTGTCC
Vimentin	CGTCCACACGCACCTACAG	GGGGGATGAGGAATAGAGGCT
Col1a1	GCTCCTCTTAGGGGCCACT	CCACGTCTCACCATTGGGG
Fn1	ATGTGGACCCCTCCTGATAGT	GCCCAGTGATTTCAGCAAAGG
TGF-β1	CTCCCGTGGCTTCTAGTGC	GCCTTAGTTTGGACAGGATCTG
α-SMA	GTCCCAGACATCAGGGAGTAA	TCGGATACTTCAGCGTCAGGA
P21	CCTGGTGATGTCCGACCTG	CCATGAGCGCATCGCAATC
β-actin	TTCGTTGCCGGTCCACACCC	GCTTTGCACATGCCGGAGCC

**Table 2:** Antibodies.

Antibody	Dilution	Catalog Number	Vendor
E-Cadherin	1:1000	3195	Cell Signaling Technology
P21	1:1000	64016	Cell Signaling Technology
CTGF	1:1000	A11067	ABclonal
Fn1	1:1000	A16678	ABclona
Beta Galactosidase Polyclonal	1:100	15518-1-AP	Proteintech
TGF-β1	1:1000	3711	Cell Signaling Technology
γ-H2AX	1:1000	9718	Cell Signaling Technology
cGAS	1:1000	A23846	ABclonal
STING	1:1000	A21051	ABclonal
GAPDH	1:1000	5174	Cell Signaling Technology

## Data Availability

All data needed to evaluate the conclusions in the paper are present in the paper. Additional data related to this paper may be requested from the authors.
